# Annotations

**Published:** 1899-09-09

**Authors:** 


### Annotations.

### Sick-Room Infection in Typhoid Fever.

Speaking at Portsmouth Dr. Herbert Peck drew attention to the fact that typhoid fever is much more frequently caught in the sick-room than is sometimes thought. The knowledge that this form of fever is very frequently carried by water, and the fact that many of the most interesting and best known epidemics of the disease have been instances of water-borne or milk-borne typhoid, have undoubtedly tended to divert attention from other modes of infection which are by no means uncommon, and Dr. Peck's observations as to the comparative frequency with which the disease is caught in the sick-room are of much value. We are so much accustomed to see nurses attending enteric patients without coming to any harm, that we are perhaps too apt to overlook the fact that their immunity is all a matter of training, and that if they do silly things typhoid fever is very definitely infectious from case to case, as Dr. Peck shows it to be among the untrained public. He gives a series of 206 cases, among which, after careful inquiry as to possible causes, it seemed probable that in 28 cases the origin of the disease was infection in the sick-room. This is equal to 13·5 per cent. of the cases investigated. Dr. Peck also concludes that sick-room infection is much commoner in the small and often crowded houses of the poor than in the larger houses of the well-to-do, which, indeed, is what one would naturally expect to be the case. He suggests as an explanation of many of the cases to which he refers, that they may be due to the practice which obtains among the poor of retaining soiled linen in use for a considerable time. He considers that, under the conditions usually met with in small houses, opportunities are afforded for the drying of portions of the excreta of the patients, and the diffusion through the air of the sick-room of the typhoid bacilli and possibly of their spores. Those, he says, who have noticed the comparatively bulky motes in a sunbeam will understand how this may occur, and he suggests that in a larger proportion of cases than is suspected typhoid fever is due to what has been called "aerial infection," but what he thinks might more appropriately be termed "sick-room infection."

### Water for Sanitary Purposes.

The great waste of water which goes on during the dry season is becoming a serious matter, especially in view of the fact that what is being so recklessly used for garden and other purposes is not crude river water, but has undergone a careful and expensive process of filtration so as to render it fit for drinking purposes. If we cannot put a stop to the present waste it may become necessary to consider the question of providing a common unfiltered water for common, rough, sanitary, and other purposes. A double water supply is a great evil in many ways, one of which is the cost of the double plant; but when we consider the great expense which is gone to at the present time, and the still greater expenditure which is proposed for the sake of obtaining a pure water, and how large a proportion of the total supply is used for purposes in regard to which absolute purity is no object; and especially when we consider that of crude river water, which is amply good enough for many purposes, we have an unlimited quantity close at our doors, to be had in any amount just for the pumping, the question does arise whether for use in central London, say within a mile of the river, a duplicate supply might not be a cheaper way out of the difficulty than some of the plans which have been proposed. Unfortunately, at present it is not in the central districts that the waste chiefly occurs. There can, however, be but little doubt that in the more crowded parts an absolutely unstinted use of water for the washing of streets, the cleansing of courts, and the flushing of sewers would be one of the most effectual methods for lessening the evils associated with prolonged drought, and a time might come when it would pay better to make use of river water for this purpose, even at the cost of having a duplicate service, than to fetch water from long distances, and, after submitting it to elaborate filtering processes for the sake of ensuring purity, use it for washing streets and pouring down the drains. It is a question of expense. At present it seems cheaper to use pure water for sanitary purposes, but it may not always be so, especially if it is to be used freely.

### Collecting Banks.

How is one to intercept the sixpence or so that even the humblest of the working class might be able to lay by, and keep it from being frittered away in some useless, even if not mischievous fashion? That is a point which often troubles workers among the poor. Even the savings banks will not take in less than a shilling, and though penny banks do most useful work, there is always the bother of going to the bank with one's humble amount. It hardly seems worth while to make a special journey to deposit sixpence; the instinct of saving must be pretty well developed before one will do that. And it is the starting of the habit of saving that is hard, not the maintaining of it. With a view to teaching the rudiments of this habit, some thoughtful people have started collecting banks. The people do not go to the bank; the bank goes to the people. Very often this work is done by lady rent collectors, who, when once they get on terms of some intimacy with their tenants, find it possible to persuade them to have just a little more than the rent ready, and to put it into the bank. It begins often almost as a compliment to the collector, a concession to the fancies of a lady who is taking a kindly interest in them. But when once the value of the plan is found out, when the odd sixpences, or even coppers, come back in a comparatively large sum to buy winter clothing, or to pay the expense of illness, the depositors begin to see the matter in another light, and appreciate the advantage of thus putting away something for a rainy day. It is only when the money is drawn out for some special purpose that the depositors begin to see what a good thing the bank is, and then, as we have said, they are anxious to put more away in a safe place, where one may keep it free from that temptation to spend it upon useless things, which besets the poor as well as the rich. One thing, however, is essential—that the collector is punctual. If she misses the day on which she is expected, the chances are ten to one that the sixpence has gone elsewhere. A use for it, wise or the reverse, has been found. It is only in the prompt and immediate withdrawing of it from income that the chance lies of converting it into capital.

